# Premature Birth and Developmental Programming: Mechanisms of Resilience and Vulnerability

**DOI:** 10.3389/fpsyt.2020.531571

**Published:** 2021-01-08

**Authors:** Femke Lammertink, Christiaan H. Vinkers, Maria L. Tataranno, Manon J. N. L. Benders

**Affiliations:** ^1^Department of Neonatology, University Medical Center Utrecht, Utrecht University, Utrecht, Netherlands; ^2^Department of Psychiatry, Amsterdam Neuroscience, Amsterdam UMC, Vrije Universiteit Amsterdam, Amsterdam, Netherlands; ^3^Department of Anatomy & Neurosciences, Amsterdam Neuroscience, Amsterdam UMC, Vrije Universiteit Amsterdam, Amsterdam, Netherlands

**Keywords:** prematurity, stress, hypothalamus-pituitary-adrenal axis, autonomic nervous system, large-scale brain networks, epigenetics, resilience

## Abstract

The third trimester of pregnancy represents a sensitive phase for infant brain plasticity when a series of fast-developing cellular events (synaptogenesis, neuronal migration, and myelination) regulates the development of neural circuits. Throughout this dynamic period of growth and development, the human brain is susceptible to stress. Preterm infants are born with an immature brain and are, while admitted to the neonatal intensive care unit, precociously exposed to stressful procedures. Postnatal stress may contribute to altered programming of the brain, including key systems such as the hypothalamic–pituitary–adrenal axis and the autonomic nervous system. These neurobiological systems are promising markers for the etiology of several affective and social psychopathologies. As preterm birth interferes with early development of stress-regulatory systems, early interventions might strengthen resilience factors and might help reduce the detrimental effects of chronic stress exposure. Here we will review the impact of stress following premature birth on the programming of neurobiological systems and discuss possible stress-related neural circuits and pathways involved in resilience and vulnerability. Finally, we discuss opportunities for early intervention and future studies.

## Introduction

The third trimester of pregnancy represents a sensitive phase for infant brain plasticity, as a series of fast-developing cellular events, such as synaptogenesis, neuronal migration, and myelination regulate the development of neural circuits (1). Throughout this period of growth and development, the human brain is highly susceptible to stress exposure. Very preterm infants are born with a neurobiological immature system and are precociously exposed to stressful procedures during weeks to months in the Neonatal Intensive Care Unit (NICU). The excessive and prolonged exposure to stress during NICU admission can exceed the infant's natural regulatory capacity, threatening the allostatic balance of the infant, and might permanently alter neuroendocrine, autonomic, cardiovascular, and neural responses (2), leading to persisting mental morbidity throughout the lifespan (3, 4).

Along with increased survival in extremely preterm born infants (EP; gestational age <28 weeks) due to continued progress in perinatal care (5, 6), the rates of dysfunction in the area of mental health and behavior have remained unchanged or even worsened during the last decades (7–10). With an increased risk for a wide spectrum of psychiatric disorders, the preterm phenotype is primarily represented by deficits in attention, executive functioning, and emotional symptoms. Importantly, whilst preterm birth is associated with a higher prevalence of psychiatric disorders, a large proportion of children remain relatively unaffected [e.g., (11)].

Here we will review the impact of stress following prematurity on the programming of neurobiological systems. We begin by giving a short overview of the different types of stressors observed in postnatal stress research, followed by the typical development of autonomic, endocrine, and top-down regulatory systems in the fetal period. We then turn to evidence that postnatal stress following prematurity has short- and long-term effects on brain development. Lastly, we discuss possible mechanisms by which postnatal adversity increases the risk for social and affective problems following prematurity. Throughout this review, we call attention to critical gaps and unanswered questions and make suggestions for future research elucidating the mechanisms linking postnatal stress, neurobiology, and future social and affective development. Where appropriate, we focus on providing evidence from human postnatal studies; however, we rely on prenatal and/or animal studies where investigations of critical questions in preterm individuals are lacking.

## Sources of Stress After Premature Birth

In the current review, we define stressors as any “*real or interpreted threat to the physiological or psychological integrity of an individual that results in physiological and/or behavioral responses*” [(4), p. 508]. In full recognition of the fact that there are numerous categorizations and types of stressors, including the administration of synthetic corticosteroids (e.g., dexamethasone) and its detrimental impact on postnatal development [e.g., (12)], for the purpose of this review we divided postnatal stressors into physical, environmental, and maternal stimuli or events. It is important to note that the effects of neonatal stress might be confounded by prenatal factors causative of preterm birth, such as vascular disease and infections (13), and hence both mechanisms may coexist. Also, the abrupt loss of intrauterine neurotrophic support following preterm birth could have deleterious effects on developmental programming, with reported damage to oligodendrocytes in the developing nervous system [for a review see (14)]. These prenatal factors are both highly relevant but are beyond the scope of the current review.

### Physical Stress

Physical stress of repetitive procedural pain occurs routinely in extremely preterm neonates who are admitted to the intensive care unit. Preterm infants and neonates show an increased physiological and behavioral sensitivity toward painful procedures, as their pain transmission and modulation are still underdeveloped [for a review see (15)]. Due to a disbalance between afferent excitatory neurotransmitters and the descending inhibitory neurotransmitters, this hypersensitivity to pain is exacerbated in preterm infants (16, 17). There are currently two main categorizations of pain-related stressors: (1) acute procedural, (2) and acute prolonged (18). Acute procedural stress is triggered by a specific noxious stimulus (19), such as a heel stick, whereas acute prolonged stress represents a longer time duration with a distinguishable beginning and expected endpoint, such as mechanical ventilation or surgery (20).

Although we currently only defined physical neonatal stress as painful procedures and interventions; medical complications, such as hypoxia, infections, and inflammation, could also be considered as extremely stressful for preterm-born individuals. Hence, the current description of physical stress is not exhaustive.

### Environmental Postnatal Stress

The effects of the nursery environment on the preterm infant, such as sound levels and nursing interventions, have become an area of concern for research. Studies showed that noise levels often exceed the American Academy of Pediatrics-recommendation of 40–45-dB (21), with NICU sounds ranging from 50 to 90 dB (22). Continuous loud noise has deleterious physiological effects on preterm infants and induces stress behaviors. More specifically, excess auditory stimulations have been associated with decreased oxygen saturation, increased heart rate and blood pressure, and alterations in sleep-wake state [for a review see (23)]. To reduce these unfavorable environmental factors, recent effort has been put into investigating the effects of single-family rooms vs. open bay units [for a review see (24) and section Opportunities for Early Intervention].

Physicians and nurses have rated routine caregiving events in the NICU as stressful to preterm infants (25). Indeed, common nursing interventions, such as diaper changes, noise, and light, can elicit similar stress-like responses as with invasive procedures [e.g., salivary cortisol levels, crying, heart rate (26, 27)]. However, to date, the impact of caregiving-related stress on the developing brain is not thoroughly researched.

### Maternal Care

Preterm infants generally experience atypical maternal care while admitted to the NICU, whilst maternal behavior toward their preterm infant plays a crucial role in the early regulation of the infant's stress responses (28). Both physical and emotional closeness might become obstructed, subsequently increasing feelings of separation and negatively impacting mother-infant attachment (29, 30). Interestingly, previous studies found that both mother's mental well-being and the physical conditions of the infant contribute independently to the degree of maternal attachment (31). More specifically, preterm infants may be regarded as less “rewarding social partners,” as their neurological immaturity negatively impacts their social responsiveness [e.g., less time in alert state (32–34)]. In other words, preterm infants might be less responsive to parental cues and show more negative expressions, which might negatively impact the quality of mother-infant interaction [for a review see (35)]. In turn, due to the circumstances, mothers of preterm infants tend to spend less time holding, talking to, and looking at their premature infant compared to mothers of full terms (36).

## Pre- and Post-Natal Brain Development of Stress Systems and Brain Networks

**Figure 1:**
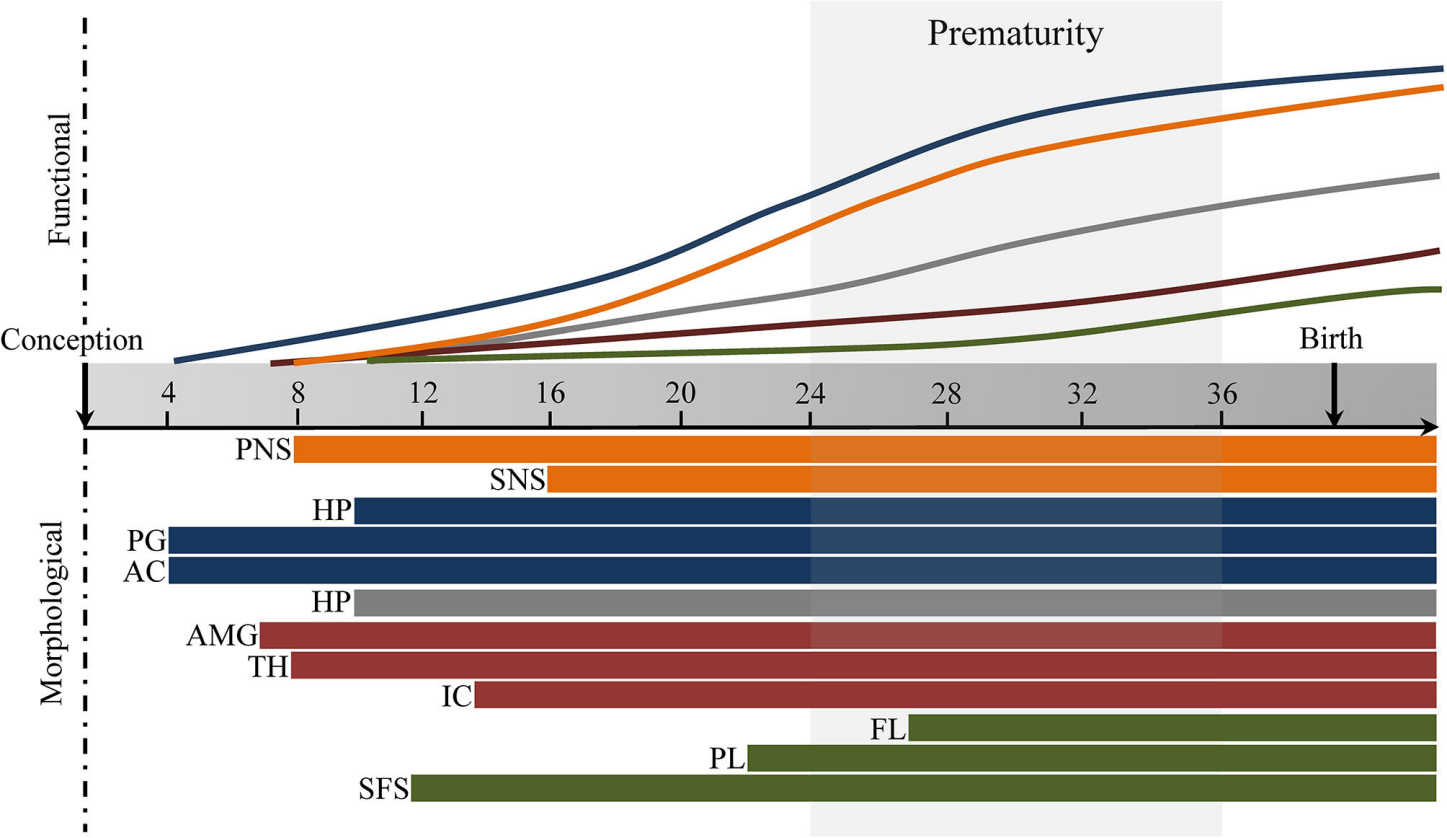
Fetal development of stress-response and stress-regulatory brain networks for illustration purposes. Autonomic Nervous System (orange), Hypothalamic-Pituitary-Adrenal axis (blue), Salience Network (red), Executive Control Network (green). PNS, parasympathetic nervous system, SNS, sympathetic nervous system, HPT, hypothalamus, PG, pituitary gland; AC, adrenal gland; HP, hippocampus; AMG, amygdala; TH, thalamus; IC, insular cortex; FL, frontal lobe; PL, parietal lobe; SFS, superior frontal sulcus. Timing based on literature reviewed under section Pre- and Post-natal Brain Development of Stress Systems and Brain Networks. Default Mode Network (gray).

Fetal brain development is characterized by large maturational changes in volume, as well as changes in microstructural and functional connectivity, which has broad implications for future development (37). These maturational changes are regulated by complex molecular and cellular processes, such as neurogenesis and neuronal migration, synaptogenesis, and axonal growth [for an overview of the spatial progression, the reader is referred to (38)]. The endocrine and autonomic stress-systems are fundamentally shaped during the fetal period. Here we will give a short overview of the spatiotemporal changes involved in the development of the autonomic nervous system (ANS), hypothalamic-pituitary-adrenal axis (HPA-axis), and stress-related brain networks, and their role in the fetal stress response (see Figure 1).

### Fetal Development of the Autonomic Nervous System (ANS)

The most immediate response to a stressor is modulated by the autonomic nervous system (ANS)—through its parasympathetic and sympathetic functions—which plays an important role in maintaining physiological homeostasis. The sympathetic discharge, during a “fight-or-flight” response, is accompanied by stimulation of the sympatho-adrenomedullary system, which is pivotal for rapid changes in physiological state, such as increased heart rate and blood pressure by catecholamine–induced excitation of the cardiovascular system (39, 40), whereas parasympathetic activation modulates the sympathetic system and restores the body to a restful state.

During the third trimester, the fetal ANS is changing rapidly (41). The maturational changes in the ANS are pivotal for the successful adaptation of the newborn to extrauterine life. The vagal nerve plays an important role in the parasympathetic regulation of autonomic functioning and subsequent socio-emotional function (42). There are two major components of the parasympathetic nervous system (PNS), namely, the unmyelinated vagal fibers [dorsal nucleus of the vagus (DMNX)] and the myelinated vagal system (nucleus ambiguus). At 9 weeks of gestation, the DMNX is distinguishable from the caudal brainstem, with the first differentiation into two subnuclei (i.e., dorsomedial and ventral) at 13 weeks. At 15–21 weeks, the subnuclei of the DMNX become more clearly visible, and the cytoarchitectonic differentiation of the DMNX is largely completed by 25 weeks (43, 44). Importantly, the phylogenetically primitive unmyelinated vagal nerve does not have many functions prior to birth, as it facilitates immobilization to deal with environmental challenges.

The nucleus ambiguus system develops later in fetal development, as it heavily depends on myelination. The nucleus ambiguus system is the primary vagal inhibitory pathway, facilitating an active vagal brake, modulating cardiac output by regulating inhibitory vagal control (45). Neurons in the nucleus ambiguus first appear at 8–9 weeks of gestation, with differentiation of neuronal subgroups appearing at 10 weeks of gestation (46). The number of myelinated vagus fibers increases rapidly from 24 weeks of gestation until the first year postpartum, indicating maturation of the parasympathetic branch of the ANS (47, 48). Advanced gestational age is accompanied by increased fetal heart rate variability (HRV) (49); these findings are in line with a rapid maturation of cardiac neuroregulatory activity and especially increasing parasympathetic tone in the last prenatal period (>32 weeks of gestation) (50).

ANS functioning is not solely mediated by the (un)myelinated vagus nerve, autonomic state is also regulated by sympathetic functioning. However, the maturation of the sympathoneural branch is less well-described. It is theorized that the sympathetic system develops before the NA, but after the DMNX (47). In line with this, research does indicate a steady maturational increase with growing fetal age from the late second into the early third trimester [~16–28 weeks of gestation (51, 52)].

Throughout most of gestation, the medulla (i.e., inner layer of adrenal gland) is not recognized as a distinct structure. At a later stage of development, after the first postnatal week, the adrenal medulla starts to form. Importantly, it takes 12–18 months for the medulla to become adult-like (53).

The fetal development of autonomic control is complex and difficult to disentangle, and important maturational milestones are reached during the transitional period from the second into the third trimester (see Figure 1). It is likely that exposure or experiences of stress during those important milestones have a potent effect on ANS development and function, which emphasizes the possible detrimental effects of preterm birth on the programming of ANS and subsequent stress-regulation [*see paragraph on autonomic nervous system (ANS)*].

### Fetal Development of the Hypothalamic-Pituitary-Adrenal Axis (HPA-Axis)

Activation of the Hypothalamic-Pituitary-Adrenal axis (HPA-axis) results in the secretion of glucocorticoids, i.e., cortisol in humans, from the adrenal cortex (39), which acts on several organ systems to mobilize energy reserves. The emerging fetal HPA-axis undergoes large shifts in maturation and organization during the prenatal period. The fetal hypothalamus could be longitudinally subdivided into three zones, namely the *midline, core*, and *lateral* zones (54, 55). Early gestation (9–14 weeks of gestation) is distinguished by differentiation of the lateral hypothalamic zone, leading to the formation of the lateral hypothalamic area (LHA) and the perifornical hypothalamus. The hypothalamic *core* is differentiated around the second trimester, 18–33 weeks of gestation. The late second until third trimester (24–33 weeks of gestation) is characterized by advances in structural maturation of the periventricular (or *midline*) zone, followed by differentiation in (1) the suprachiasmatic [i.e., circadian clock under the strong influence of light/dark input (56)], (2) arcuate [i.e., sensor to modulate cortisol release (57)], and (3) paraventricular nuclei [i.e., promotes corticotrophin releasing hormone (CRH) and vasopressin (AVP) (58)]. These maturational changes extend into the third trimester. Around the postnatal period (immediate after birth), the major hypothalamic structures are clearly differentiated and resemble an adult-like form (54, 55).

The fetal pituitary gland seems to mature before the adrenal cortex. More specifically, the kidney-shaped anterior lobe of the pituitary gland, which is connected to the hypothalamus, starts to form from Rathke's pouch by 4–5 weeks of gestation. The first 12 weeks of gestation are characterized by major cellular differentiation (59), and by 21 weeks a further distinction can be made between the long and thin stalk region of the pituitary and the posterior lobe (60). Although the pituitary gland matures until the third postnatal month, after which it appears to be adult-like (61), fetal adrenocorticotrophin hormone (ACTH) is detectable by 8–10 weeks of gestation, peaking between the first and second trimester, after which it declines late in gestation (62).

Starting at the 4th weeks of gestation, the adrenal cortex (i.e., outer layer of adrenal gland) begins to form, and the morphology remains relatively stable after 10–12 weeks of gestation. Research suggests that the fetal adrenal cortex is unable to synthesize cortisol between 16 and 22 weeks of gestation, as the 3ß -hydroxysteroid dehydrogenase (3ß -HSD; converts pregnenolone to progesterone) enzyme is not expressed before the start of the third trimester (63). Hence, these findings indicate that the fetus is able to adapt to environmental changes and to maintain homeostasis, through glucocorticoid secretion, after 23 weeks of gestation. However, there is not much consensus on when exactly the fetal adrenal cortex is able to synthesize cortisol. More specifically, at 30 weeks of gestation, the fetal adrenal cortex resembles the elementary form of the adult adrenal cortex [(64), see (65), for review], and some studies suggest that the fetal adrenal cortex is unable to produce cortisol *de novo* until then (66), but instead uses the abundant placental progesterone. This would indicate that in absence of placental progesterone, the fetus might be unable to produce cortisol before 30 weeks of gestation.

The developmental trajectories of the HPA-axis that are established during the prenatal period could have lifelong consequences for future development (see Figure 1). Preterm-born infants are neuroendocrinologically immature, and their NICU stay associated with (multiple) stressful events might disturb the central regulation of HPA-axis. Therefore, prematurity might be characterized by the inability to maintain homeostasis in the face of acute stress [see *Hypothalamic-Pituitary-Adrenal axis (HPA-axis)*].

### Fetal Development of the Stress-Related Neural Networks

There are three core neural networks that are implicated in the central response of stress, namely the (1) default mode network [DMN; which includes the posterior cingulate cortex (PCC), hippocampus, and parahippocampal cortex, amongst others (67)], the (2) salience network [SN; which includes the dorsal anterior cingulate cortex (ACC), frontoinsular cortex, amygdala, and several other (sub)cortical structures (68)], and (3) executive control network [ECN; which includes dorsolateral prefrontal and parietal regions (69)]. The ability to dynamically shift neural resources within these large-scale networks is theorized to facilitate adaptive responses to stress, and alterations in these networks possibly underlie phenotypic abnormalities (70). Interestingly, nodes within these networks start to develop in the fetal period, showing large morphological and functional changes throughout gestation. Below we will give a short overview of the typical maturational changes in the embryonic and fetal period (see Figure 1).

#### Default Mode Network

The *hippocampus*, a core node of the DMN, plays an important role in the regulation of the stress response due to its high expression of glucocorticoids and mineralocorticoids, thereby exerting negative feedback on the HPA-axis (71). As early as 9 weeks post-conception, four distinct hippocampal layers can be distinguished: intermediate zone, ventricular zone, hippocampal plate, and marginal zone (72). An unfolded hippocampus, along the medial surface of the temporal lobe, is present at 13 weeks of gestation. Throughout the following weeks, infolding of the hippocampus into the temporal lobe start as the dentate gyrus and cornu ammonis develop into an interlocking C shape. By 18–21 weeks of gestation, the hippocampus shows morphological maturity similar to that of the adult brain (73). The absolute volumes increase linearly from 14 to 22 weeks of gestation, with, relative to other brain regions, a faster growth from 14 to 17 weeks, ]but a slower growth from 18 until 22 weeks (73, 74). The dentate gyrus develops latest, showing a mature cytoarchitercute after 34 weeks of gestation (72). Compared to other brain structures, the hippocampus seems to be one of the earliest developing brain regions in humans. Importantly, the morphological development of the *PCC* and *parahippcampal gyrus*, both important components of the DMN, are not well-documented.

Important connections of the adult DMN are already present in the fetal period. For instance, some short-range pathways between the hippocampus and cortico-cortical regions, i.e., entorhinal cortex, are established as early as 19 weeks of gestation (75). Functional studies reported that from 19 weeks onwards, connectivity of the PCC became increasingly negative, which according to the authors, might serve a foundational role in the establishment of large-scale neural networks (76). Similarly, older (>35 weeks of gestation) but not younger fetuses showed a more synchronized positive functional connectivity between the PCC and medial PFC, and negative connectivity to the lateral prefrontal and parietal regions (77). Although the DMN becomes more synchronized across the first 2 years of life and achieve adult-like structures at the end of the first year, including increased connectivity between the PCC and hippocampus, the network is still rather immature in neonates (78–80). In sum, much of the foundation of the DMN is laid down in the early fetal and neonatal period. With the unfolding of several neuromaturational processes, disturbances in normative brain development, including an adverse extra-uterine environment, has likely far-reaching consequences. More studies are needed to elucidate the structural and functional milestones of the DMN and the impact of neonatal stress.

#### Salience Network

The *amygdala*, a core node of the SN, is a key component of the limbic system and is commonly implicated in emotional and behavioral regulation. This structure shows large morphological changes during fetal development. Differentiation of the amygdala nuclei continuous from the embryonic through the fetal period and neurogenesis is completed by birth (7.5–34 weeks gestation). More specifically, at 12 weeks of gestation migration of the neurons to the lateral amygdaloid nucleus are visible (81), and all major nuclei are formed by 15 weeks. The amygdala appears to be fully mature and functional at birth (81–83), and its connections are laid down early in gestation. Despite the absence of myelin, at a very early stage (i.e., 13–22 weeks of gestation) the amygdala establishes the first connections to several areas of the cortex (84, 85) with the appearance of association white matter fibers, such as the uncinate fasciculus [i.e., a major white matter fiber tract connecting the anterior temporal lobe and the amygdala to the lateral orbitofrontal cortex to the inferior frontal cortex (86)], appearing at around 15 weeks of gestation.

Although structural amygdala connectivity appears early in fetal development, these connections are predominantly short-range, with long-range tracts becoming more evident by term (87). Similarly, the functional connections of the amygdala stabilize early in fetal development. Late second and early third trimester (21st−26th weeks of gestation) are dominated by occipital and temporal connections, with a substantial increase in functional connectivity between the frontal and temporal lobes later in gestation [29–37 weeks of gestation (88)].

The *thalamus* is another core node of the salience network, which is a region that is strongly connected to the amygdala and involved in the regulation of stress, amongst others (89). From 8th week on, thalamic neurons show intensive morphological changes, with projection from the spinal cord to the thalamus. From 10 to 14 weeks of gestation, neuronal differentiation into several thalamic nuclei begins (90). Neurogenesis in the posterior medial thalamus extends into the late first and early second trimesters of pregnancy. By 26 weeks' gestation, the characteristic layers of the thalamus are visible, with obvious similarities to the adult brain. Thalamocortical pathways to the subplate neurons are evident at 17 weeks of gestation, but the thalamic projections to the cortical plate continue to develop later during the fetal period (24–32 weeks) (84, 85, 87, 91), reaching adult-like connections at 34 weeks of gestation.

It is well-known that subcortical structures demonstrate earlier maturation than the cerebral cortex. However, the *insular cortex*, also described as the “center of salience processing” (92), is among the first macroscopical structures that can be identified in the human fetal cortical development. Afif et al. (93) described the morphological stages of insular sulci and gyri maturation, with the first sulcus appearing at 13–17 weeks of gestation. Around 27–28 weeks of gestation, all insular sulci and gyri are in place and its structure is similar to its adult-like form. More specifically, by the end of the third trimester, the insula can be divided into two parts, the anterior insula (i.e., comprises three short gyri), and the posterior insula (i.e., comprises two long gyri). Radial migration pathways, between the ventricular zone and superior temporal region, were observed at 15 weeks of gestation, continuing to grow in number and thickness until 20 weeks of gestation (94). Further, by 26 weeks of gestation, migration pathways are regressing, and by 31 weeks of gestation the insular neuronal migration is fully completed. Importantly, similar connectivity is observed between 31 and 40 weeks of gestation, and it seems that several pathways observed in the fetal period (e.g., insular-parietal and insular-temporal pathways) are similar to those in adults.

The fact that several core nodes of the salience network mature before birth emphasizes the importance of early fetal development for functioning of the salience network. To date, there are only a few studies directly investigating early development of structural and functional connectivity within the salience network. One study did report synchronous activity of the anterior insula with anterior cingulate cortex in neonates, although quite primitive (95). Interestingly, however, while the neonatal brain consisted of large locally connected clusters, 1- and 2-year olds demonstrated more sophisticated distributed topology. Further, enhanced connectivity between the anterior insular and long-range prefrontal cortices and anterior cingulate cortex seemed adult-like in 1-year olds (with only a few changes in 2-year olds) (95). In line with this, other studies do suggest that connection strength increase with age, but only moderately, leading to still premature network topologies at the end of the first year (80) and second-year (96). Interestingly, recent studies were able to identify so-called “hubs,” which are highly connected regions, in the fetal period. Both the temporal lobe (97) and the insular cortex (76, 98) were found to be already highly connected before birth. In sum, key nodes of the salience network seem, on a morphological level, adult-like at birth, but the topological features of fetal brain network remain underdeveloped (see Figure 1). Changes in typical fetal development, such as preterm birth, might precociously impact the architectural characteristics of the immature SN.

#### Executive Control Network

Brain regions that serve a high-order function, such as the *dorsomedial prefrontal cortex* (DMPFC) and dorsal *posterior parietal cortex* (DPPC), which are all core nodes of the ECN, mature latest. The corticogenic events occur at different rates in different regions, and some cortical areas start to differentiate earlier in gestation than others. For instance, studies consistency showed a maturational lag in the frontal cortical regions, as the neural migration [i.e., as indicated by peak fractional anisotropy [26–30 weeks of gestation]] of the frontal lobe is preceded by the parietal lobe (21–25 weeks gestation) [e.g., (99, 100)]. Some studies suggest that the superior longitudinal fasciculus (SLF), a white matter tract connecting the superior parietal and superior frontal lobes and extending to the dorsal premotor and dorsolateral prefrontal regions, is completely absent in the fetal brain (84, 101). However, a recent study was able to reliably visualize the SLF at 26 weeks, suggesting that the SLF may start to develop in the second trimester, and continue to develop throughout gestation (102). In line with this, short- and long-range corticocortical association pathways could be observed at 22 weeks of gestation, and become more prominent throughout gestation (103).

Short-range cortico-cortical tracts emerge prior to gyrification in regions where sulci will later develop. The cortical plate starts to form in the human telencephalon around 7–10 weeks of gestation (104). The timing of the different types of sulci occurs hierarchically. Primary sulci start to appear as early as 10 weeks of gestation and continue to develop until the 28th week of gestation (105, 106). This is followed by the development of secondary and tertiary sulci. All the primary and most of the secondary sulci are believed to be present by 34 weeks of gestation (106, 107), whilst tertiary sulci appear around term [36–41 weeks of gestation (108)]. A core node of the ECN, the *superior frontal sulcus*, emerges between 22 and 24 weeks of gestation (109–111), and become clearly visible from ~27 weeks onwards (105, 106, 112).

Although the ECN is observable in fetuses and neonates (see Figure 1), it is still in a premature form at the end of the first postnatal year (79). However, the fetal period constitutes a time of vast development, and a study on functional connectivity found large differences in the ECN between younger and older fetuses. More specifically, they reported that older fetuses (i.e., 34 weeks of gestation) showed increased connectivity between the prefrontal areas and the parietal cortex, compared to younger fetuses (i.e., ~27 weeks of gestation) (76). In sum, using structural and functional connectivity and graph-theoretical analyses, studies were able to provide preliminary evidence for the emerging ECN in fetuses, highlighting the importance of the establishment of ECN nodes to the effect of functional and structural injuries typically sustained during premature birth. Importantly, the large variability in spatiotemporal development of nodes seem to indicate a developmental sequence starting from the DMN, to the SN, and finally the ECN, as the (sub)cortical nodes implicated in the DMN and SN appear to be maturing during the beginning of the first trimester, as opposed to the relatively delayed maturation of cortical nodes implicated in the ECN.

## Neonatal Stress and Brain Development

More than 50 years ago, researchers expressed their concerns regarding the possible detrimental effects of neonatal stress on physiology and behavior, stating that early experience in animals, such as handling or electric shock, lead to changes in corticosterone response and emotionality [e.g., (113–115)]. Rodent studies gave rise to a large body of experimental studies reflecting the importance of early neonatal stress in regulating brain development (116, 117). The studies that have been conducted thus far found quite consistently that, similar to animal studies [e.g., (118)], exposure to neonatal stress is associated with alterations in several structures. For instance, using the Neonatal Infant Stressor Scale [NISS; cumulative stress score including physical and environmental stressors (25)], researchers found an association between high stress and decreased frontal and parietal brain volumes, as well as alterations in white matter microstructure in the temporal lobes in preterm born infants at term equivalent age (119). Similarly, the number of invasive procedures was associated with reduced white matter and subcortical gray matter maturation in preterm neonates. Moreover, greater invasive procedures were independently associated with reduced total brain volume, white matter diffusivity, and maturation of subcortical gray matter (e.g., thalamus, basal ganglia) (49, 120). Interestingly, Brummelte and colleagues could distinguish a period of increased vulnerability, namely early procedural pain [i.e., before scan 1 (median 32.1 weeks of gestation)] had a stronger association with white matter maturation compared to later stress (around term equivalent age), whereas subcortical gray matter showed sustained sensitivity toward neonatal stress (120). These results are in line with findings from another study, where they reported that functional and structural connectivity patterns were affected following exposure to early (i.e., from birth to scan 1), but not late neonatal stress [i.e., scan 1 to scan 2 [term-equivalent age]]. More specifically, early neonatal stress (e.g., heel lances, central line insertion) was associated with weaker structural (121) and functional (122) connectivity between the right insula and limbic system, and in the thalamocortical pathways. However, some inconsistencies remain, as researchers also reported no association between neonatal stress and hippocampal growth (123). With some ambiguity, these studies converge to reveal a developmental period of increased sensitivity to stress, subsequently affecting stress-regulatory networks. However, there have been no further studies to confirm the possible detrimental effects of postnatal stress on large-scale brain networks, and the possible increased risk of future social and affective functional impairment.

The adverse effects associated with exposure to postnatal physical stress appear to extend beyond the relationships observed in the neonatal period. For instance, a higher number of invasive procedures during NICU admission was related to abnormalities in white matter microstructure (e.g., superior white matter), as reflected by an increase in radial diffusivity at age 7 (124). Procedural pain was also observed to be related to abnormal maturation of brain volumes at school age, including the hippocampus, amygdala, thalamus, striatum, globus pallidus, and cerebellum (125, 126), and lower cortical thickness (127). Studies using magnetoencephalagraphy (MEG) found that differences in spontaneous gamma- to alpha-band oscillations in preterm-born school-age children were predicted by the number of invasive neonatal procedures, which, as suggested by the authors, might be attributed to alterations in thalamorcortical connectivity (128, 129). Hence, the abnormal maturation observed in the neonatal period seems to persist into childhood, affecting brain regions implicated in the regulatory capacity to future stressors (see Table 1 for an overview).

**Table 1:** Neonatal stress and neurobiological systems.

**References**	**Population**	**Time of assessment**	**Sample size (N)**	**Stress measure**	**Outcome**
**Brain development**					
Brummelte et al. (120)	Infants (born 24–32 weeks)	32 and 40 weeks	86	Number of invasive procedures: early (birth-scan 1) and late (scan 1-scan 2).	Greater invasive procedures: ↓ white matter FA, ↓ subcortical gray matter NAA/choline. Effects dependent on timing stress.
Chau et al. (125)	Children (born <32 weeks)	8 years of age	57	Number of invasive procedures during the stay in the NICU	Greater invasive procedures: ↓amygdala volume, ↓ thalamus volume. Stress × COMT ↓ hippocampal subregional volume
Doesburg et al. (128)	Children [born extremely preterm [24–28 weeks], very preterm [28–32 weeks], and full-term]	8 years of age	54	Number of invasive procedures during the stay in the NICU	Greater invasive procedures: atypical spontaneous neuromagnetic activity (*only in extremely preterm born children*)
Duerden et al. (123)	Infants [born very preterm [<33 weeks]]	32 and 40 weeks	138	Number of invasive procedures during the stay in the NICU: categorized into two groups	Greater invasive procedures: no association with hippocampal growth
Duerden et al. (121)	Infants [born extremely preterm [24–28 weeks] or very preterm [29–32 weeks]]	32 and 40 weeks	155	Number of invasive procedures: early (birth-scan 1) and late (scan 1-scan 2)	Greater invasive procedures: ↓ lateral thalamus volume, ↓ metabolic growth (NAA/Cho), ↓ FA corpus callosum, posterior white matter, cingulum, and fornix. (*only in extremely preterm born children in combination with early stress*)
Kozhemiako et al. (129)	Children [born extremely preterm [24–28 weeks], very preterm [29–32 weeks], and full-term]	8 years of age	100	Number of invasive procedures during the stay in the NICU	Greater invasive procedures: atypical spontaneous neuromagnetic activity (*only in extremely preterm born children*)
Ranger et al. (127)	Children [born very preterm [27–32 weeks]]	8 years of age.	42	Number of invasive procedures during the stay in the NICU	Greater invasive procedures: ↓ cortex thickness (e.g., frontal, parietal, and temporal regions)
Ranger et al. (126)	Children [born very preterm [27–32 weeks]]	8 years of age	42	Number of invasive procedures during the stay in the NICU	Greater invasive procedures: ↓ cerebellar volumes
Schneider et al. (49)	Infants [born very preterm [<30 weeks]]	29, 31, and 40 weeks	51	Number of invasive procedures during the stay in the NICU	Greater invasive procedures: ↓ growth thalamus, basal ganglia, total brain volumes
Smith et al. (119)	Infants [born very preterm [<30 weeks]]	Term equivalent age	44	Neonatal Infant Stressor Scale: during stay in the NICU or until term equivalent age	Greater number of stressors: ↓ frontal and parietal diameter, and ↓ interhemispheric connectivity temporal lobes
Tortora et al. (122)	Infants [born very preterm [<33 weeks]]	Term equivalent age	46	Number of invasive procedures: categorized into four groups	Greater invasive procedures: ↓ connectivity thalami—bilateral somatosensory cortex, ↓ connectivity insular cortex—ipsilateral amygdala/hippocampus
Vinall et al. (124)	Children [born very preterm <33 weeks)	7 years of age	50	Number of invasive procedures during the stay in the NICU	Greater number of stressors: ↓ white matter FA
**ANS function**					
Goffaux et al. (130)	Children [born very preterm [<33 weeks], and full term]	7–11 years of age	26	Total number of days spent in the NICU and total numbers of days spent under mechanical ventilation: categorized into two groups	Greater invasive procedures: no changes in heart rate and pain sensitivity in high-stress group in response to conditioning cold stimulation
Grunau et al. (131)	Infants [born very preterm [<33 weeks]]	32 weeks	136	Number of invasive procedures from birth until time of assessment	Greater invasive procedures: ↓ autonomic reactivity in response to blood collection
**HPA axis function**					
Brummelte et al. (132)	Children [born extremely preterm [<28 weeks], very preterm [<32 weeks], full term]	7 years of age	129	Number of invasive procedures from birth until term equivalent age	Greater invasive procedures: ↓ basal cortisol (study day and at home)
Grunau et al. (133)	Infants [born extremely preterm [<28 weeks], very preterm [<33 weeks], and full term]	8 months	76	Number of invasive procedures from birth until term equivalent age	Greater invasive procedures: ↑ sustained basal cortisol (*only in extremely preterm born infants*)
Grunau et al. (134)	Children [born very preterm [<33 weeks] and full term]	7 years of age	128	Number of invasive procedures from birth until term equivalent age	Greater invasive procedures: ↓ hair cortisol (*stress × NFKBIA effect, only in boys*)
Provenzi et al. (135)	Infants [born very preterm [<33 weeks] and full term]	3 months of age	90	Number of invasive procedures during the stay in the NICU	Greater invasive procedures: ↓ cortisol reactivity to still-face procedure
**Epigenetics**					
Chau et al. (136)	Children [born very preterm [<33 weeks] and full term]	7 years of age	111	Number of invasive procedures during the stay in the NICU	Greater invasive procedures: ↓SLC6A4 methylation (*only in children with COMT Met/Met genotype*)
Fumagalli et al. (137)	Infants [born very preterm [mean of 30 weeks]]	Birth and NICU discharge	56	Principal component analysis on number of invasive procedures	Greater invasive procedures: ↑ delta SLC6A4 methylation
Montirosso et al. (138)	Infants [born very preterm [<33 weeks] and full term]	Birth and NICU discharge	78	NICU stay: difference score between birth and NICU discharge	↑ delta SLC6A4 methylation at discharge than at birth
Provenzi et al. (139)	Infants [born very preterm [<33 weeks] and full term]	Birth and NICU discharge	88	Number of invasive procedures during the stay in the NICU: categorized into two groups	Greater invasive procedures: ↑ delta SLC6A4 methylation

*↑ increase(d); ↓ decrease(d)*.

These findings are supported by evidence from fetal stress models demonstrating that full-term and preterm neonates exposed to prenatal stress showed alterations in brain development. More specifically, prenatal stress (e.g., maternal anxiety/depression) was associated with reductions in region-specific gray matter volume [i.e., PFC, temporal lobe (140)], alterations in white matter microstructure (e.g., amygdala, limbic system) (141–143), and reduced functional and structural connectivity between the amygdala, limbic, and frontal regions in infants (144–146). The effects of prenatal stress on brain development have been extensively reviewed elsewhere [see (147–149)].

Although changes in previously described brain regions have been consistently implicated in a wide range of behavioral problems in preterm born individuals [e.g., (150–155)], it remains elusive whether alterations in brain development might modulate the relationship between neonatal stress and future affective and social functioning.

## Mechanisms Underlying Later Life Resilience and Vulnerability Following Prematurity

### Autonomic Nervous System (ANS)

*Ex utero* third trimester development often leads to alterations in normal autonomic development in extremely preterm infants, which is essential for respiratory and cardiovascular homeostasis (156). General maturation of the ANS is often assessed using indices of heart rate, blood pressure, and respiratory rate. Studies showed an impaired autonomic maturation in preterm born neonates, as reflected by dampened sympathetic (e.g., low-frequency HRV) and parasympathetic (e.g., high-frequency HRV) tone (41, 50, 157, 158). Thus, far only one study investigated the sympatho-adrenomedullary system (SAM), with results indicating elevated sympathoadrenal tone (as indicated by increased levels of catecholamines) in preterm-born children (159). This increased release of catecholamines could be attributed to the reduced parasympathetic inhibition, as mentioned previously.

The autonomic development is altered during the neonatal period. Accordingly, an increasing body of evidence suggests that ANS dysfunction following prematurity persists into infancy (160, 161), childhood [(162, 163); but not all (164)], adolescence (165), and adulthood (166). This prolonged abnormal maturation of ANS functioning could be attributed to the amount of neonatal stress. More specifically, research showed that greater exposure to neonatal stress was related to dampening of ANS reactivity (130, 131).

Changes in central autonomic regulation in typically developing individuals limit the capacity to adequately respond to environmental changes, and have previously been implicated in psychiatric disorders (167–169). Consistent with this, ineffective vagal modulation has been implicated in dysfunctional emotion regulation in toddlers [e.g., (170)], children [e.g., (171)], and adults [e.g., (172)]. Interestingly, similar findings were observed in preterm infants, namely preterm born infants' degree of respiratory sinus arrhythmia (RSA) was positively associated with their social competence, whereas lower (mean) heart rate was associated with less behavioral problems and greater social competence (173). These findings indicate that functional deficiencies of the vagus, and more specifically the phylogenetically newer ventral vagal complex (VVC; i.e., part of the nucleus ambiguus and suppresses robust emotional reactions), might underlie difficulties in emotion regulation in preterm-born individuals (see Table 1 for an overview).

Although no further studies investigate the relationship between ANS functioning and outcome, these findings conform to the framework of the *polyvagal theory*, proposed by Porges (45, 174–176), which states that alterations in vagal tone and reactivity, and thus parasympathetic regulation, possibly lead to the development of psychiatric disorders in preterm-born individuals. The framework articulates three phylogenetic stages that underlie different behavioral responses, all associated with a distinct autonomic subsystem: (1) social communication [e.g., emotion (via ventral vagal complex)], (2) mobilization [e.g., fight-flight responses [via sympathetic-adrenal system]], and (3) immobilization [e.g., tonic immobility (via unmyelinated vagus)]. Alterations in these distinct subsystems possibly underlie the behavioral problems observed in preterm individuals. However, further research is needed to delineate the effects of preterm birth, and postnatal stress, on autonomic control and subsequent longitudinal brain and behavioral development.

### Hypothalamic-Pituitary-Adrenal Axis (HPA-Axis)

A considerable array of research has found that neonatal adversity impacts neuroendocrine development. Studies reported, for instance, that more invasive procedures were associated with lower cortisol responses to a stressor [below 33 weeks post-conception (133); for a review see (177)]. Also, hyporeactivity to socio-emotional stress, as measured with the Face-to-Face Still-Face (FFSF) procedure (i.e., assesses socio-emotional regulation by rating negative emotionality, social engagement, and avoidance behavior), has been linked to the number of invasive procedures during NICU admission in 3-month old preterm infants (135). In children born very preterm, greater exposure to neonatal pain-related stress was associated with higher basal cortisol levels at 8 months (133) and 18 months (178), but lower basal, diurnal (132) and cumulative (hair) cortisol (134) at age 7–8 years. It is well-recognized that chronic stress can lead to downregulation of cortisol production [e.g., (179)], thereby reducing the detrimental effects of glucocorticoids. These alterations in HPA-axis functioning seem to persist into adulthood. More specifically, studies showed both decreased and increased HPA-axis responses (i.e., cortisol and ACTH) following acute stress, when compared to full-term controls (180, 181). These mixed results might be due to the developmental timing at which neonatal stress occurs, including age of onset, duration, and severity, affecting the effects of concurrent stress on the dynamically changing HPA-axis (*see paragraph on fetal development of the hypothalamic-pituitary-adrenal axis*).

The different developmental stages seem to be mirrored by a shift between hypo- and hyper-reactivity of the HPA-axis, with postnatal stress possibly altering the set-point of HPA-axis functioning in preterm-born individuals. This observation highlights the fact that both the type and magnitude of the stress-responses largely depends on the timing of the stressor. Moreover, exposure to postnatal stress at different points in the development of the HPA-axis might exhibit a different impact. Indeed, rodent studies showed that “early” maternal separation (i.e., 3–4 postnatal days) was associated with a hyper-responsivity to stress later in life, while “late” maternal separation (i.e., 7–8 or 11–12 postnatal days) showed an effect in the opposite direction (182, 183). These findings are in line with studies indicating that synthesis of CRH receptors is regionally distinct and age-specific. More specifically, CRH receptor density in rodents is highest during early postnatal days (i.e., 2–9), with CRHR1 mRNA levels increase to a maximal of 300–600% of adult levels (184). Perturbation of the profound changes in CRF system, due to extreme and chronic stress, might have long-lasting consequences for development. Hence, these critical developmental processes are extremely complex, and dependent on the timing of exposure, postnatal stress might have differential consequences. However, to date, this explanation for the mixed results of HPA-functioning in preterm born individuals remains speculative, and research is needed to delineate the possible time-specific effects of postnatal stress following preterm birth.

Surprisingly, altered HPA-axis functioning and its impact on (brain) development in ways that increase susceptibility to later stress-related disorders have not been extensively studied in preterm born individuals (see Table 1 for an overview). Thus far, higher basal cortisol in preterm born infants has been associated with poorer mother interactive behavior, as well as more problems in terms of emotional reactivity, anxiety, depression, and attention, amongst others (185). Similar findings were observed in preterm-born children, that is, increased HPA-reactivity was linked to more problems with attention, emotional reactivity, anxiety, depression, and negative mother-child interactions (186). Hence, converging evidence does suggest that HPA-axis functioning is a key mediator of developing psychopathology. Although the structural and functional development of neural networks is tightly linked to HPA-axis functioning, such that early life adversity sensitizes hippocampal-amygdala responses to acute stress (187, 188), to date, there are no studies that explored neural circuitry associated with HPA-dysfunction in preterm born individuals.

In sum, postnatal stress following prematurity lead to long-lasting changes in HPA-functioning, which, in turn, is associated with problem behavior (e.g., emotional reactivity, anxiety). Differences in HPA-functioning might be attributed to the timing of postnatal exposure, although extensive research is needed to disentangle the neurobiological mechanisms involved. Importantly, there is currently little research as to whether the altered patterns of HPA-axis functioning in the context of prematurity and postnatal stress are permanent, what epigenetic pathways might underlie the contradicting findings (*see paragraph on epigenetic pathways*), and the potential of prevention following postnatal interventions such as skin-to-skin contact (*see paragraph on possibilities for early intervention*).

### Epigenetic Pathways

There is increasing evidence for the role of genetic and epigenetic variation in long-term effects of early life stress (189). It is suggested that epigenetic markers are developmentally sensitive to the quality of the pre- and post-natal environment and that early adversity produces lasting epigenetic modifications (190–192). Studies on the so-called “early-life programming” of the epigenetic regulation of gene transcription, have mainly focused on the serotonin transporter, due to its polymorphisms and role in mediating early stress and later life mental health, and glucocorticoids (GR), due to its negative feedback control on stress responsivity [for a review see (193)]. Importantly, most studies have focused on candidate genes related to serotonin and glucocorticoid functioning. Below we will review evidence from postnatal studies, but we will rely on prenatal studies where investigation in preterm individuals is lacking.

There are a few studies that investigated the influence of postnatal stress on *SLC6A4* [i.e., a gene encoding the serotonin transporter (*5-HTT*)] promoter methylation. Studies reported an association between greater postnatal stress and lower *SLC6A4* methylation in preterm-born infants (138, 139) and school-aged children with the *COMT 158 Met/Met* genotype (136). Importantly, authors suggested that *SLC6A4* promoter methylation could not be attributed to preterm birth *per se*, rather, high levels of postnatal stress exposure altered the transcriptional functionality of *5-HTT* (139, 194). In turn, greater *SLC6A4* methylation predicted poorer stress-regulation in response to the still-face procedure at NICU discharge (194). Additionally, methylation at discharge was associated with greater negative emotionality and suboptimal socio-emotional regulation (137, 194). Thus far, only one study investigated the possible moderating role of *SLC6A4* methylation on the relationship between NICU-stress and later brain development. More specifically, authors reported that preterm infants exposed to greater stress showed higher *SLC6A4* methylation, which in turn was associated with reduced anterior temporal lobe (ATL) volumes (137).

Extensive scientific literature has repeatedly reported that changes in glucocorticoid receptor methylation (*NR3C1*) play a pivotal role in the regulation of the HPA-axis and the endocrine response to stress (195). Indeed, infants exposed to third-trimester prenatal stress, as measured by maternal emotional state, showed increased methylation of the *NR3C1* gene (196, 197). Additionally, Oberlander and colleagues found that increased methylation of the *NR3C1* was in turn associated with increased HPA-axis reactivity. Even though *NR3C1* is tightly linked to stress vulnerability and resilience, studies investigating the epigenetic changes in *NR3C1* following prematurity is limited. Few studies did investigate the role of prematurity in *NR3C1* methylation, and findings were in line with studies on prenatal stress [for a review see (193, 198)], that is, preterm infants exposed to an adverse postnatal environment influenced *NR3C1* methylation (199). Specifically, increased methylation of glucocorticoid receptor gene was observed in the first 4 days following preterm birth. However, findings remain inconsistent as studies demonstrated both decreased and increased DNA methylation of *NR3C1* in high-risk preterm infants [i.e., scoring high on Neonatal Intensive Care Unit Network Neurobehavioral Scale (NNNS) or more medical problems] compared to low-risk preterm infants (200, 201).

Several other genes have been found imperative for the regulation of HPA-axis function, including the *FKBP5* gene, which exerts an inhibitory role on GR signaling by modulating hormone-binding affinity (i.e., the strength of binding interaction) (202, 203), and the 11beta-hydroxysteroid dehydrogenase type 1 and 2 (*11*β*-HSD*), functions as a dehydrogenase which degrades cortisol to cortisone (*11*β*-HSD2*), and catalyzing the conversion of inactive cortisone to active cortisol (*11*β*-HSD1*) [for a review see (204)]. So far, only one study investigated *FKBP5* gene transcription in relation to prematurity. Piyasena et al. (205) showed that preterm born infants had markedly lower methylation at *FKBP5* compared to term-born infants. Importantly, these differences in *FKBP5* methylation were resolved at 1 year of age. In line with this, a longitudinal epigenome-wide association study (EWAS) identified a total of 1,555 sites with significant differences in methylation in preterm born infants, but the majority of these differences did not persist into adulthood (206). Hence, these studies question whether the effects of prematurity and postnatal stress on DNA methylation persist across the life course.

*11*β*-HSD1* is seen in the adult central nervous system and has previously been shown to influence HPA-axis regulation (207, 208), whereas *11*β*-HSD2* has a major central role in developmental programming due to the high expression in placenta and fetal tissue (209). As the function of *11*β*-HSD* seems to reflect protection from the deleterious consequences of glucocorticoid overexposure, it has been suggested that *11*β*-HSD* might function as a potential pathway in early life stress exposure and later outcome (210, 211). Studies indeed showed that prenatal stress was associated with downregulation of placental *11*β*-HSD2* gene encoding (212, 213), as well as lower and greater methylation of *11*β*-HSD2*, respectively (214, 215). In turn, in rodents, *11*β*-HSD2*^−/−^ selectively determined programming of anxiety and depressive-like adult behavior (204, 211, 216). Similarly, *11*β*-HSD1*^−/−^ mice showed elevated basal corticosterone levels and exaggerated responses to stress (207, 208, 217). Increased levels of placental β*-HSD1* mRNA were observed in mothers exposed to prenatal stress (218, 219), leading to increased glucocorticoid transport to the fetus. Although these findings could theoretically be extrapolated to a preterm population, as the assumed changes in *11*β*-HSD* fail to protect the immature neurons from premature stress exposure, further investigation will be required to determine the degree to which changes in *11*β*-HSD* are prevalent in preterm born individuals.

Only recently studies began to enhance our understanding of epigenetic changes following prematurity. Converging evidence suggests that preterm born individual show profound changes in glucocorticoid and serotonin transporter gene transcription, with some studies suggesting that these alterations can be specifically attributed to postnatal stress. Other genes involved in the regulation of HPA-axis, such as *FKBP5* and *11*β*-HSD*, have not been studied extensively. Importantly, epigenetic changes following prematurity might be non-persistent. Nonetheless, more studies are needed to further delineate the epigenetic changes following prematurity and the specific role of postnatal stress. There are several theoretical and methodological challenges in the field of behavioral epigenetics in preterm born individuals, including heterogeneity of the population (e.g., gestational age), lack of prospective longitudinal and epigenome-wide studies, small sample sizes, and inadequate control of confounders (e.g., race), amongst others.

### Disruptions in the Neural Equilibrium

It is well-known that stress induces large-scale neural modulations and that extreme and prolonged stress trigger long-lasting changes in network balance. Both the salience network (SN) and the executive control network (ECN) are implicated in the adaptive regulation of stress, and these complex networks already originate in the fetal period (*see paragraph on Fetal development of the stress-related neural networks*). In the face of environmental challenges, via increased catecholamines, the SN is supposedly upregulated, facilitating increased vigilance and attentional reorienting, and autonomic-neuroendocrine control (220). On the contrary, the ECN, which is implicated in cognitive control processes and decision-making, is downregulated following stress (221). It is theorized that in the aftermath of stress, resources are allocated to the ECN, downregulating the SN (70). Disruptions in this neural equilibrium, possibly due to morphological alterations in prefrontal (222) and hippocampal (223) neurons following chronic stress, have been implicated in the pathogenesis of Post-Traumatic Stress Disorder (PTSD) (224, 225), depression (226, 227), anxiety (228, 229), bipolar disorder and schizophrenia (230, 231), amongst others.

#### Emotion Processing and Executive Functioning

A large number of studies have described a behavioral phenotype in preterm-born individuals constituting problems in the area of socio-emotional and executive functioning [for further details please see (232–234)], two higher-order cognitive phenotypes implicated in stress regulation (235, 236). The neural mechanisms underlying these behavioral phenotypes appear to be altered in preterm-born individuals. Although there are currently no studies investigating the role of postnatal stress on these stress-regulatory neural mechanisms, a growing number of studies does recognize the pivotal role of large-scale neural networks, rather than region-of-interest (ROI) based approaches, in preterm-born individuals.

Resting-state network studies showed an altered coupling between the SN and default mode network (DMN). The DMN comprises the medial prefrontal, posterior cingulate, precuneus, and bilateral angular gyrus (237), and exhibits low-frequency activity at rest, and has been proposed to be related to self-referential mental activity, including task-unrelated imagery and thoughts and self-reflection in preterm born individuals (238). Studies consistently reported a hypo-connectivity between the amygdala, mPFC, posterior cingulate cortex (PCC), anterior insula (AI), and the precuneus (pC) in preterm-born infants at term equivalent age (239), adolescents (240), and adults (241, 242). As suggested by the authors, the negative connectivity between the SN (i.e., AI/amygdala) and DMN (i.e., PCC/pC) could indicate an overactive inhibitory function of the PCC in modulating the left amygdala, greatly affecting their emotion processing (241). In line with this, a study reported that variability in the connectivity between the amygdala and other regions was predictive of greater internalizing symptoms at 2 years (239). Also, alterations in white matter microstructure involved in the SN and other networks, including the thalamus, inferior fronto-occipital fasciculus, and inferior/superior longitudinal fasciculus have been associated with internalizing symptoms in preterm born children aged 9–16 (243).

Preterm children also showed structural alteration in the anatomical organization of the cortico-basal ganglia-thalamo-cortical pathway (CBGTC). Especially connections between the thalamus, putamen, globus pallidus and caudate nucleus were weaker (151). These SN-specific nodes are disrupted in psychiatric diseases [e.g., PTSD, obsessive-compulsive disorder (OCD), schizophrenia (244)], dissociating multiple interconnected mental operations, such as processing affective content, decision making, and attention (245). Studies also reported significant smaller amygdala volumes in preterm-born infants at term equivalent age, compared to term born infants, and this altered amygdala development has been linked to maladaptive fear-processing (as measured by Unpredictable Mechanical Toy episodes) (246). A key limbic tract, the uncinate fascicle [i.e., temporo-amygdala-orbitofrontal network (247)], critical for social-emotional functions (86, 248), showed significant white matter reductions in preterm-born individuals [children; (249); adolescents (250)]. At present, it would be helpful to have a clearer understanding of how these neural patterns vary as a function of postnatal stress.

These limbic-cortical pathways are not only involved in socio-emotional behavior but also play a pivotal role in higher-order behavioral control. Studies repeatedly reported significant alterations in white matter microstructure, including the cingulum (i.e., connection between anterior cingulate cortex, dorsolateral prefrontal cortex, and inferior parietal lobe), fronto-occipital fascicles (i.e., bridging frontal-temporal-parietal-occipital lobe), fornix, corpus callosum, and superior longitudinal fasciculus, amongst others (153, 251–255). These white matter indices have been directly linked to alterations in executive functioning, that is, lower executive functioning was related to reductions in several white matter microstructure (e.g., inferior-fronto-occipital fascicles, cingulum, and superior longitudinal fasciculus) [Wisconsin Card Sorting Test (256); Test of Everyday Attention for Children (TEA-Ch) (257); Child Behavior Check List (CBCL) (153); Delis-Kaplan Executive Function Systems (D-KEFS) (258)]. Importantly, one study was unable to find an independent effect of preterm birth on white matter microstructure, and authors emphasized that postnatal factors, such as the degree of stress (i.e., days of mechanical ventilation), grossly impact postnatal brain development (259).

The alterations in connectivity are also observed on a functional level. Specifically, studies found that preterm born adults, compared to controls, showed increased activity in the middle temporal/occipital gyrus, posterior cingulate gyrus, and precuneus [go-/no-go task; (260); verbal fluency task (261)], which replicates previous findings (262). Additionally, during oddball trials (i.e., “odd” stimuli to control for low-frequency no-go stimuli), preterm-born young adults displayed attenuated brain activation in a fronto-parietal-cerebellar network. More recent studies disentangled the neural underpinnings of proactive vs. reactive cognitive control in preterm-born adults. Using the Not-X continuous performance test (CPT), authors reported (1) hypo-activation between the frontal pole and anterior cingulate gyrus, as well as the posterior cingulate gyrus and precuneus, and (2) hyper-activation between the posterior cingulate gyrus and precuneus, and the right lateral occipital cortex and angular gyrus (255). In other words, authors showed that preterm born adults exhibited more reactive behavioral control, rather than proactive. Recent research started to reveal the importance of these functional patterns, as well as the decoupling between the ECN and DMN, for the development of cognitive control (263) and emotion-regulation (264).

In sum, preterm born individuals show profound alteration in large-scale brain networks involved in emotion regulation and executive functioning, the SN and ECN, respectively. These changes possibly underlie individual differences in stress-sensitivity, as both large-scale networks, and its behavioral phenotype, have been implicated in adaptive stress responses (70). As mentioned previously, the DMN, SN, and ECN start to develop during gestation. Although these networks are still immature at birth, the formation of important pathways during gestation gives rise to potential points of vulnerability. The degree to which postnatal stress might underlie these extensive network changes in preterm born individuals remains elusive.

## Opportunities for Early Intervention

Inadequate treatment of stressors in preterm infants have previously been associated with short- and long-term alterations in brain and behavior, greatly impacting their ability to maintain homeostasis (*see paragraph on neonatal stress and brain development*). This highlights the importance of adequately and promptly assessing stress in the newborn, and gave rise to the Newborn Individualized Developmental Care and Assessment Program (NIDCAP) (265, 266), which is a technique that uses detailed observations of infant behavior to provide caregivers and parents with recommendations on how to minimize stress. Developmental care theories postulate that one should actively observe the infant, during several caregiving procedures (e.g., collection of a blood sample), to assess the infant's efforts of self-regulation in response to stress. Based on such observations, both clinicians and families can make adjustments to optimize and adapt the traditionally delivered newborn intensive care to the infants' current needs. These adjustments can include interventions developed to increase self-regulation in the preterm infant. In full recognition of the fact that there are numerous interventions aimed at preventing or reducing postnatal stress, including the possible protective effects of fetal neurosteroids such as allopregnanolone, and its inhibiting properties in relation to the HPA-axis [(267, 268); for a review see (269)], for the purpose of this review, we decided to focus on non-pharmacological interventions as several analgesics have the potential to adversely impact the developing postnatal brain by altering neuronal processing [e.g., (270, 271)].

Skin-to-skin contact, also called kangaroo care, is a promising intervention possibly reducing infants' stress during NICU admission. For instance, research showed that preterm born infants exhibited lower basal stress levels (i.e., autonomic responses) during kangaroo care when compared to regular care (272, 273), as well as lower stress reactivity when exposed to physical stress (274–277), and improved white matter microstructural development (278). Kangaroo care also seems to affect HPA-axis functioning, as indicated by reduced saliva cortisol levels when exposed to a period of kangaroo care (279), as well as lower salivary cortisol reactivity at 1 month (280). However, kangaroo care seems to not bring about sustained reductions in salivary cortisol (280–282). Interestingly, research did show sustained effects on autonomic control. More specifically, one study found that kangaroo care accelerated the maturation of vagal tone between 32 and 37 weeks of gestation, as indicated by increased amplitude of RSA, positively affecting autonomic control (283). As suggested by the authors, kangaroo care might exert developmentally sensitive effects and should be administered during an appropriate developmental window to alter the maturational trajectories of systems that are currently developing. In other words, the timing of the intervention is pivotal for achieving optimal levels of physiological maturation. Although kangaroo care showed some positive effect on preterm infants' physiological stability and maturation, the degree to which these effects are long-term remains elusive.

Although inconclusive, there is some evidence for the positive effects of kangaroo care on attachment behavior and parental stress levels. More specifically, kangaroo care positively affected mother's mood, reduced parental stress levels, increased parental affiliative (e.g., touch) and attachment behavior when compared to standard care [e.g., (177, 272, 284, 285)]. These changes in parental care and behavior might, in turn, have positive effects on the preterm infant's stress-regulatory capacities [e.g., (286, 287)], which provide preliminary support for changing the biological organization of the stress system in preterm infants through parental-driven interventions.

There is growing evidence to promote not only the use of kangaroo care, but also the use of music, massage, co-bedding, and Family Nurture Interventions in extremely preterm infants (288–297). Despite the use of different measures to assess stress and pronounced differences in intervention, results from these studies largely indicated improvement in HPA-axis functioning and autonomic control, as indicated by lower cortisol levels and increase autonomic stability following the intervention. These auditory and tactile interventions can be viewed as environmental enrichment, possibly stimulating cortical plasticity and attenuating the stress response in preterm infants (298). Indeed, recent studies found encouraging, but preliminary, evidence for increased microstructural maturation at term-equivalent age for preterm infants exposed to music during their NICU stay (299), as well as a greater maturation of cardiac function (296). In the long-term, both Family Integrated Care, i.e., infant care provided by families by enhancing parental support and education (300), and Family Nurture Interventions, i.e., promotes calming interaction between mother and infants, seem to have a sustained positive effect of behavioral outcome, with more robust self-regulation skills and less negative emotionality at 18–21 months and 4–5 years of age, amongst others (297, 301, 302). Further research is warranted into the exact neurobiological pathways underlying the relationship between tactile and auditory interventions and preterm infant stress-regulation.

There are several reasons to hypothesize that single-family rooms (SFR) will reduce infant and parental stress, increase parental involvement, and subsequently improve infants' outcome. Nonetheless, scientific evidence on the benefits of SFR, vs. open bay, is at this point mixed and questions whether the change from open bay to SFR is justified [for a review see (24)]. Some studies reported positive benefits of private rooms, including less physiological stress, improved neurobehavioral development, and better long-term outcomes in preterm infants (303–305). On the contrary, studies also reported potential adverse effects in relation to SFR, that is, increased maternal stress (306, 307), altered infant cerebral development, and worse neurodevelopmental outcome (308), and no effects on infant salivary cortisol reactivity (309). Together, these findings suggest that consideration of the design and environment is important for the health and well-being of preterm infants admitted to the NICU, including noise control, parent-infant closeness, and parental involvement. However, much uncertainty remains regarding the design of the NICU environment, how much or what type of sensory stimulation would optimize brain development in preterm infants. It is important to realize that both neurosensory deprivation, a consequence of SFR, and neurosensory overexposure, a consequence of open ward, might be maladaptive (310).

In sum, a range of aspects of the physical environment is pivotal for stimulating development of the preterm infant. Research showed beneficial effects of parent-infant bonding, sensory stimulation, and the use of private family-rooms on mitigating postnatal stress in preterm infants. Moreover, these interventions seem to promote infant self-regulation, by adequately utilizing their neurophysiological modulatory system to safeguard oneself from excessive over-stimulation and arousal. Hence, appropriate tactile and auditory stimulation seems sufficient to induce improvement in self-regulation. Nonetheless, results are not consistent, and the degree to which these improvements are sustained remains inconclusive. Also, several methodological challenges, such as lack of standardization, possibly introduce confounding effects.

## Concluding Remarks

In summary, the reviewed literature suggests that stress has both proximate and long-lasting detrimental effects on brain and behavior in preterm-born individuals. A key question is whether there are, so-called, “sensitive” periods of pre- and post-natal development during which stress-regulatory mechanisms are established. Indeed, research indicates that not only the HPA-axis and ANS are formed during the embryonic, fetal, and postnatal period, also large-scale brain networks implicated in the central response of stress including the DMN, SN, and ECN start to develop early on. This window of increased vulnerability offers an important clue for the cellular impairment that might underlie stress-sensitivity and long-term outcome in preterm born individuals.

**Figure 2:**
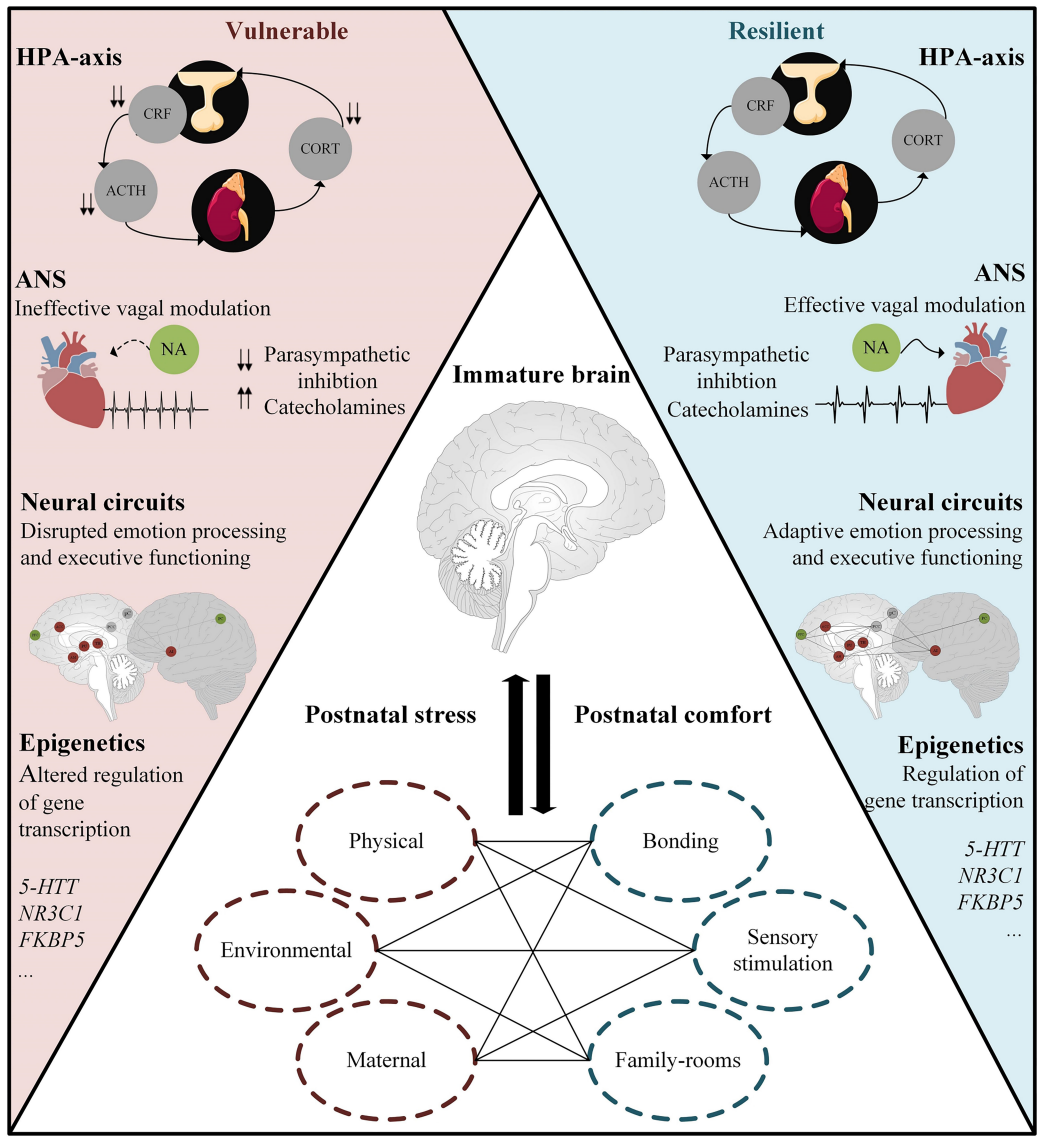
Resilient functioning in preterm born individuals exposed to chronic postnatal stress might be facilitated by; the ability to regulate and dampen stress responsivity; effective vagal modulation; homeostasis in large-scale neural networks underlying emotion processing and executive functioning; and adaptive regulation of gene transcription. Middle panel: Postnatal factors influencing brain maturation and, in turn, the onset/development of stress-related disorders such as anxiety and depression. ACTH, adrenocorticotropic hormone; Cort, cortisol; CRF, corticotropin-releasing factor; HPA-axis, hypothalamus–pituitary–adrenal axis; ANS, autonomic nervous system; NA, Nucleus Ambiguus; *5-HTT*, serotonin transporter; *NR3C1*, glucocorticoid receptor; *FKBP5, FK506* binding protein 5 and acts as a co-chaperone that modulates glucocorticoid receptor activity.

A few studies have emerged to investigate the effects of “early” vs. “late” stress. Postnatal work indeed demonstrated that early stress has differential and long-lasting effects on brain development, compared to stress experienced around term-equivalent age. Importantly, some studies also suggested an independent effect of postnatal stress on brain development, rather than prematurity *per se*. The current review underscores increasing evidence that postnatal stress might persistently impact later functioning in part by affecting neurobiological systems, including the HPA-axis, ANS, large-scale brain networks, and gene expression. Also, an increasing number of intervention studies found preliminary evidence for the possible beneficial effects of parent-infant bonding, sensory stimulation, and family rooms on mitigating the detrimental effects of postnatal stress. However, the reviewed evidence is far from conclusive and there is a paucity of clinical studies on the subject of prematurity and stress resilience and vulnerability. We have only started to investigate the role of postnatal stress in resilience and vulnerability to developing psychiatric disorders following prematurity. Therefore, our schematic presentations of some proposed relations (see Box 1 and Figure 2) should not be considered comprehensive, rather, it should guide future research toward possible modulating factors between postnatal stress and future resilience or vulnerability in preterm born individuals.

Box 1 **Unanswered questions.**

1. Following premature birth, how do postnatal stressor type, timing, and duration translate to changes in neurobiological systems and subsequent outcome across the life course?

2. Which (combination of) neurobiological mechanisms underlie vulnerability or resilience following postnatal stress?

3. Are neurobiological changes following premature birth (i.e., physiological, neural network, and epigenetic) indicative of maladaptive functioning or do they also occur as a result of adaptive processes in stress-regulatory systems?

4. Can targeted and individualized neonatal interventions reverse morphological and functional remodeling of neurobiological systems and lead to improved outcomes? Also, when are these interventions able to buffer the effects of postnatal stress on neurobiological development to mitigate long-term risk for affective and social functioning?

5. What is the potential of identifying individual developmental trajectories, and the subsequent early identification of who is and who is not at risk following preterm birth and chronic stress exposure?

To better understand and reduce the impact of postnatal stress and prematurity on brain development, psychopathology, and possible mechanisms, one should consider integrating several additional issues into future studies (see Box 1 for unanswered questions). First, existing research has predominantly focused on the effects of physical stressors, such as skin-breaking procedures, rather than taking into account different stressor types, including maternal care and environmental stress. As pointed out previously, there is a great dependence of stressor type in characterizing risk factors. In other words, each stressor has distinct and possibly cumulative effects on later development of brain and behavior, and failing to account for stressor type might greatly impact results. Hence, there is a great need for systematic research on the possible detrimental effects of postnatal stress. These investigations should particularly focus on the possible long-term effects of postnatal stress on later development of brain and behavior, including adolescence and adulthood.

Second, it is important to note that only a minority of preterm born individuals will develop a psychiatric disorder. It has become clear that several neurobiological pathways might react differently to neonatal stress in resilient and vulnerable individuals. However, the specific factors that interact and account for these differences are still undetermined. Behavioral studies reported that positive parental behavior is favorable and predisposes preterm individuals to stress resilience (297, 301, 302, 311, 312). On a neurobiological level, detailed analyses of factors that account for and interact with variation in several moderating and mediating factors (e.g., HPA-axis, epigenetics), in both vulnerable and resilient individuals, is lacking. Such research would benefit from prospective longitudinal studies, with a systematic assessment of postnatal stress, and the association with changes in neurobiological systems and later affective and social functioning. For instance, as stress has a global effect on brain functioning, investigating large-scale brain networks gives a greater insight into the reorganization of connectivity patterns, rather than limiting analyses to predefined regions of the brain (313). To date, it remains unclear whether and how acute stressors relate to shifts in resource allocation between large-scale brain networks, and whether specific neural patterns might underlie resilience in preterm born individuals. Most importantly, how these networks dynamically unfold as a function of stress, and whether postnatal stress might alter these dynamics, remains unanswered.

Third, the ability to “bounce-back” is not viewed as a stable trait, rather, it is viewed as malleable and easily modified by interventions (314). Certain postnatal interventions appear to be beneficial in reducing the detrimental effects of postnatal stress, particularly interventions increasing parent-infant bonding (e.g., kangaroo care). However, considerable uncertainty remains as to whether these beneficial effects persist over time, and what, if any, neurobiological systems are remodeled.

Preterm born individuals have an increased risk for developing psychiatric sequelae, and this heightened vulnerability might have its origin in the postnatal exposures. Fortunately, research investigating the role of postnatal stress on later development in preterm born individuals has gathered momentum over the past two decades, and an increasing number of studies have focused on ways to diminish the detrimental effect of postnatal stress. Although the mechanisms that lead to resilient phenotypes is far from being fully determined, the current review identified several potential factors that might facilitate an adaptive stress response in the face of adversity. An increased understanding of the neurological, physiological, and epigenetic circuitry underlying resilience in preterm born individuals might be a starting point for the development of targeted and individualized intervention and prevention programs.

## Author Contributions

FL drafted the paper. CV, MT, and MB provided critical revisions. All authors approved the final version of the paper prior to submission.

## Conflict of Interest

The authors declare that the research was conducted in the absence of any commercial or financial relationships that could be construed as a potential conflict of interest.
